# Evolution of Blind Beetles in Isolated Aquifers: A Test of Alternative Modes of Speciation

**DOI:** 10.1371/journal.pone.0034260

**Published:** 2012-03-30

**Authors:** Remko Leijs, Egbert H. van Nes, Chris H. Watts, Steven J. B. Cooper, William F. Humphreys, Katja Hogendoorn

**Affiliations:** 1 South Australian Museum, Adelaide, South Australia,Australia; 2 School of Earth and Environmental Sciences, University of Adelaide, Adelaide, South Australia, Australia; 3 School of Biological Sciences, Flinders University of South Australia, Adelaide, South Australia, Australia; 4 Department of Aquatic Ecology and Water Quality Management, Wageningen University, Wageningen, The Netherlands; 5 Western Australian Museum, Welshpool, Western Australia, Australia; 6 School of Animal Biology, University of Western Australia, Nedlands, Western Australia, Australia; 7 School of Agriculture, Food and Wine, The University of Adelaide, Adelaide, South Australia, Australia; Lund University, Sweden

## Abstract

Evidence is growing that not only allopatric but also sympatric speciation can be important in the evolution of species. Sympatric speciation has most convincingly been demonstrated in laboratory experiments with bacteria, but field-based evidence is limited to a few cases. The recently discovered plethora of subterranean diving beetle species in isolated aquifers in the arid interior of Australia offers a unique opportunity to evaluate alternative modes of speciation. This naturally replicated evolutionary experiment started 10-5 million years ago, when climate change forced the surface species to occupy geographically isolated subterranean aquifers. Using phylogenetic analysis, we determine the frequency of aquifers containing closely related sister species. By comparing observed frequencies with predictions from different statistical models, we show that it is very unlikely that the high number of sympatrically occurring sister species can be explained by a combination of allopatric evolution and repeated colonisations alone. Thus, diversification has occurred within the aquifers and likely involved sympatric, parapatric and/or microallopatric speciation.

## Introduction

Strong evidence for sympatric speciation has recently been provided in vitro [1], and the concept is well supported by theoretical analyses [2]–[7]. Often cited examples from natural systems involve the evolution of new species in relatively closed systems such as crater lakes (e.g. cichlid fishes [7], [8]), and islands (e.g. Anolis lizards [9], palms [6], [10], [11] Hawaiian spiders [12]) where there is evidence for colonisation by a single ancestral species and subsequent niche partitioning. However, even after the presence of sympatric, closely related sister species has been established, it remains uncertain whether the co-occurring species pairs have evolved in sympatry or whether the divergence of the species occurred in isolation and involved multiple invasions [13]–[16]. To distinguish between these modes of speciation a statistical approach is needed, which requires the presence of multiple sympatrically occurring species pairs. Such data sets have hitherto been lacking.

The recent discovery of communities of invertebrates in Australian subterranean aquifers that have evolved in isolation for millions of years provides a unique opportunity to evaluate the occurrence of sympatric versus allopatric modes of speciation in a natural environment.

**Figure 1:**
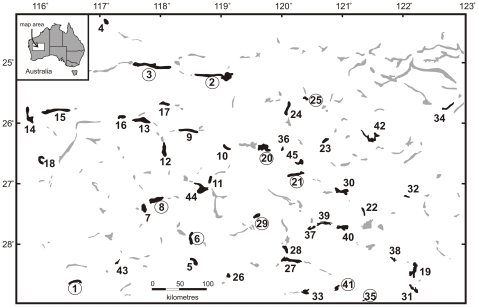
Distribution of calcrete aquifers in Western Australia. Black: aquifers where subterranean diving beetles were found; grey: aquifers not sampled or not containing diving beetles. The numbers denote aquifer localities; numbers in circles are aquifers containing sympatric sister clades. The coordinates and species composition of the aquifers are given in the supplemental information S1.

In the Late Miocene – Pliocene, (10-5 million years ago, Mya), the interior of Australia underwent aridification [17]. During this process hundreds of subterranean aquifers in calcrete limestone deposited along palaeo-drainage systems became biologically isolated [18]–[22] (Figure 1). Phylogenetic studies [21] revealed that surface species of diving beetles (Coleoptera, Dytiscidae) took refuge in these subterranean aquifers during one or more periods of extreme aridity. This resulted in the evolution of one to three species of blind, wingless (apterous), de-pigmented (stygobitic) endemic species per aquifer. The aquifers all provide similar, very stable ecological conditions [23], and thus the colonisation events can be viewed as a repeated natural speciation experiment.

The massive radiation generated by this natural experiment has only recently been uncovered. In the last 12 years 99 new stygobitic beetle species have been described from 52 isolated aquifers [24]. These species now represent, by far, the world's most diverse subterranean diving beetle fauna [25]. Each beetle species is restricted to a single aquifer, indicating the complete isolation of the system. The coexisting species all differ markedly in size and morphology ([24] and references therein), which points to the possibility that they occupy distinct niches. In most aquifers, the co-existing species appear to be descendants from distantly related ancestral lineages [21], suggesting an allopatric process of speciation. However, eleven of the studied aquifers contain sister species.

Here, as advocated by Fitzpatrick et al. [26], we investigate the biological processes that may have lead to the divergence of the sympatrically occurring sister species. We consider two hypotheses to explain the presence of sister species in the same aquifer. Firstly, the same ancestral surface species may have colonised an aquifer repeatedly at different times, for instance, during different aridity maxima [27]. In this case, the first colonising species would evolve into a new stygobitic species prior to a second invasion by the same ancestral lineage. Secondly, speciation may have occurred *within* the aquifer, after invasion of the underground habitat by a single ancestral species.

**Figure 2:**
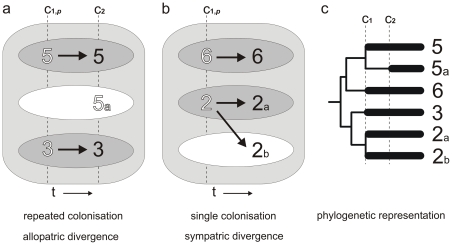
Schematic representation of the colonization models. (a and b): The outer box represents a calcrete aquifer, the ovals represent individual niches, which may get colonized with niche colonization probability *p*, C_1_ and C_2_ are colonization events. The numbers represent colonizing species that are randomly drawn out of a pool of *n* ancestral species. (c): Phylogenetic representation of the models; bold lineages evolve underground. Species 5 and 5a are sympatric sister species that evolved by repeated colonization; species 2a and 2b are sympatric sister species that diverged in the aquifer after the colonization of their ancestor species 2; species 3 and 6 independently colonized aquifers.

We use a statistical model to test the repeated colonisation hypothesis and evaluate it against the within-aquifer speciation hypothesis. The model (Figure 2, methods) predicts the fraction of aquifers containing sympatric sister species, assuming two or three colonisation events. We show that it is unlikely that the observed high frequency of co-occurring sister species pairs and triplets is the result of repeated colonisations, and that the pattern is better explained by diversification within the aquifers.

## Results

### Phylogenetic analyses

**Figure 3:**
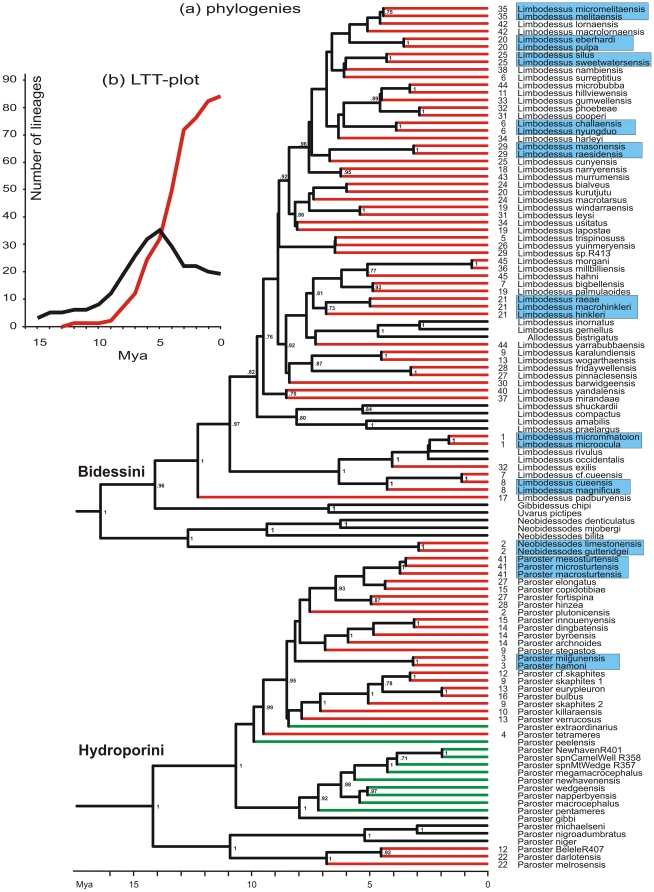
Molecular phylogeny and lineage trough time plot of dytiscid diving beetles. (a): Molecular phylogeny with sympatric sister pairs (blue boxes) shown. Red lines indicate terminal branches leading to a subterranean species. It is assumed that somewhere on the branch the colonization of the subterranean environment took place (see also Leys et al 2003). Black lines indicate surface lineages. Green lines indicate subterranean lineages from aquifers outside of the Yilgarn region. These were not used in the analyses. The numbers at the tips of the branches refer to the Yilgarn calcrete aquifer localities as indicated in Figure 1. Posterior probabilities >0.7 of the Bayesian analyses are indicated near the branches. (b): Lineage-through-time (LTT) plot for surface (black) and subterranean (red) lineages demonstrating the presence of twenty or more ancestral surface species during the major radiation of subterranean beetles 3–7 Mya.

Bayesian phylogenetic analyses (Figure 3a) demonstrate a well-supported topology of two tribes of diving beetles (Dytiscidae). These phylogenies also show nine cases of sympatric sister pairs, and two triplets of sympatric sister species. Nine of these sympatric sister clades are supported by high (1.00) posterior probability values, while the two clades that have lower support were also found with parsimony and neighbour joining analyses using PAUP* [28] and Bayesian analyses using MrBayes [29](data not shown). Parameter estimates from the Bayesian analyses are available in the supporting information S2. A lineage-through-time (LTT) plot (Figure 3b) shows that the major radiation of subterranean beetles took place 3–7 million years ago.

### Repeated colonisation model

**Figure 4:**
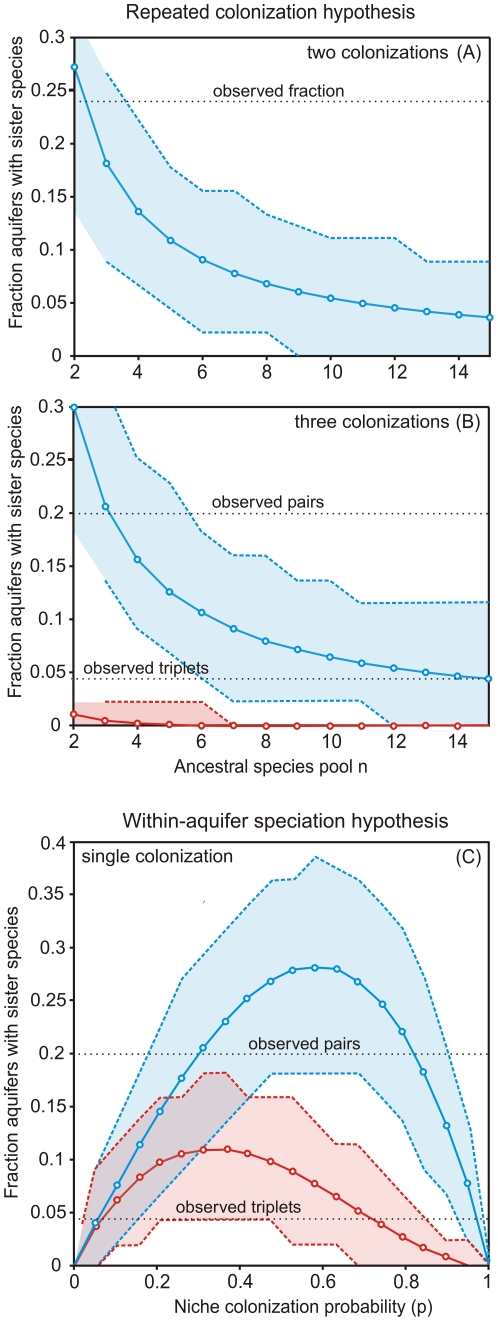
Results of colonization modeling. (a & b) Repeated colonization model. (a) The relationship of the size of the ancestral species pool and the fraction of the aquifers containing sister species after two colonization events (formula 1). An initial niche colonisation probability (p_1_) of 0.5 was used as this maximises the probability of sister pairs (see supporting information S3). The last colonisation probability (p_2_) was set to 1. The observed fraction of aquifers with sympatric sister species (11/45) is also indicated. (b): The predicted fraction of aquifers containing sympatric sister pairs (blue; formula 2) and triplets (red; formula 3) calculated based on three colonization periods and a niche colonization probability (p_1_ = p_2_ = 0.4) that maximizes the probability of pairs and triplets (see supporting information S3). Horizontal lines indicate the observed fraction of aquifers with sister pairs and triplets. The last colonisation probability (p_3_) was set to 1. (c): Within-aquifer speciation model. The relationship between the initial niche colonization probability and the predicted fraction of aquifers containing sympatric sister pairs (blue) and triplets (red) calculated with single colonizations and subsequent divergence within aquifers. Horizontal lines indicate the observed fraction of aquifers with sister pairs and triplets. The models are calculated using the observed number of aquifers with one (18 aquifers), two (16 aquifers) or three (11 aquifers) species. The shaded areas in (A–C) represent the 5% and 95% percentiles as confidence limits from 10000 randomizations. We assumed that all open niches were filled by speciation (q = 1).

We used a model (see methods) to test the repeated colonisation hypothesis in order to predict the fraction of aquifers with pairs and triplets of sympatric sister species. If we assume two distinct colonisation events, the probability of finding sympatric sister species is maximized when the initial colonization probabilities are 0.5 (supporting information S3 Figure 4). At this value, the model generally predicts a much lower number of sister pairs than observed and no triplets (Figure 4a). The observed fraction of sister pairs is within the 95 percentiles of the model outcomes if the ancestral species pool is assumed to contain less than 4 species. If we assume three colonisation events, the predicted number of pairs do not change substantially (Figure 4b), while the probability of finding triplets remains very small and only possible with very low numbers of ancestral species.

The phylogenetic analysis (Figure 3) shows that during the major radiation of subterranean species, the number of ancestral species was certainly larger than four (actually more than 20, Figure 3a and 3b). Therefore, we reject the repeated colonisation model as the only explanation for the evolution of sister species in aquifers.

### Single colonisation model

Next, we explore the most extreme alternative, i.e. whether the number of sympatric sister pairs can be explained by single colonisations and subsequent speciation within the aquifers (see methods). Here, the predicted fraction of sister species does not depend on the number of ancestral species. Note that the model now generally predicts higher values than observed. However, if the initial colonization probability is between 0.2 and 0.85, the predicted number of pairs and triplets of sister species does not differ significantly from the observed values. An initial colonization probability of circa 0.78 provides a near-accurate prediction of the fraction of aquifers with sister taxa based on the observed 9 pairs and 2 triplets out of 45 aquifers (Figure 4c). Therefore, in contrast to the repeated colonisation model, the model of single colonisation, which assumes subsequent diversification within the aquifers, is capable of predicting the observed number of sympatric sister species.

## Discussion

This study provides strong support for speciation of blind water beetles within the isolated aquifers. Under the alternative model of allopatric speciation and repeated colonization by the same ancestral surface species, the frequency of sympatric sister species would be significantly lower than observed in the field. Thus, repeated colonization alone cannot explain the high frequency of pairs and triplets of sympatric sister species in the aquifers, and therefore, at least some speciation within the aquifer needs to be invoked to explain the high frequency of sister species pairs and triplets. Especially the probability of finding two triplets of species is extremely low in the model assuming repeated colonization. However, whilst the analyses demonstrate that a majority of the sister species can be explained by speciation within the aquifer, we cannot assert that this holds for *all* 11 sympatric sister groups. In some cases, speciation could have taken place by repeated colonisation, as suggested above.

The value of a model critically depends on its assumptions. The main assumption of our model of repeated colonisation is that the ancestral species have an equal chance to successfully colonize aquifers, else the ancestral species pool will be effectively smaller. To meet this assumption the ancestral species must have had widespread and largely overlapping geographical distributions. As already noted by Darwin [30], and confirmed by other studies [31]–[37], most dytiscid water beetles indeed have very widespread overlapping distributions, are able to fly large distances and are capable of rapidly colonizing newly available habitats, such as roadside ditches, ponds or temporary streams. Moreover, intrinsic factors of species, such as size or pre-adaptations to subterranean life, could make certain species more likely colonizers than others. Although diving beetle assemblages usually consists of a number of distinct size classes [38], in the system described here only species belonging to genera that exclusively fit in the smallest size class (2–5 mm) appear to have successfully colonized the aquifers. A lineage-through-time (LTT) plot (Figure 3b) shows that prior to the major radiation of the subterranean species at least 30 ancestral species within these genera were present.

We further assumed that an aquifer can only contain a limited number of species, or niches. This is fully supported by the available data [21], [24], [39] showing that despite intensive survey work in the Yilgarn area over the last decade only up to 3 beetle species where found in each aquifer. A further assumption is that once a species has successfully colonised an aquifer it is very unlikely to subsequently colonise and diverge into another aquifer. The rationale for this is that suitable aquifers are isolated from each other by fine alluvial sediments that do not allow subterranean dispersals and above ground dispersal would be hampered by stygobiontic (eg. loss of wings, pigment, eyes) adaptations. The pattern of unique species per aquifer has also been found for several other taxa that live in these aquifers, such as Amphipoda [18] Isopoda [19] and Bathynellacea [20] and supports the long-term and near complete isolation between aquifers. Secondary divergence would only be possible when a single aquifer becomes physically fragmented.

Unlike several other speciation models [2]–[4], [6], [7], our model does not include assumptions about the genetic and ecological processes of diversification. We simply tested whether the occurrence of multiple independent sympatric sister pairs could be explained by repeated colonisation by the same ancestral lineages as a null-model, as an alternative to a process of speciation occurring within aquifers. We explored the behaviour of the repeated colonisation model by maximising the probability of finding sympatric pairs by using equal niche colonisation chances and by assuming that unoccupied niches will always be filled during the second colonisation. Even under such conservative parameter settings, allopatric speciation by repeated colonisations by ancestral lineages could not explain the observed number of sympatric sister species within aquifer.

Although our analysis provides strong support for speciation within the aquifer, we hesitate to classify these speciation events as sympatric speciation, for several reasons. First, as indicated by Butlin et al. [40] and Fitzpatrick et al. [26], [41], speciation processes should be viewed as a continuum in geographic modes with sympatric and allopatric speciation at the extremes. Second, identification of a mode of speciation for the sympatrically occurring sister species would require unwarranted speculation about the nature of reproductive barriers within the aquifers. Third, such a classification would ignore the possibility that, during the formation of the sister species groups, reproductive isolation varied over time. In this context, it is more informative to investigate the extant reproductive barriers within aquifers.

Conceivably, there are two scenarios for microallopatric/parapatric speciation. First, aquifers with a linear structure could become colonised by a single ancestral species at different localities simultaneously, while an overlap in their within-aquifer distributions is established only after a period of time has allowed the populations to become genetically and reproductively isolated. Second, some of these aquifers may become physically fragmented, e.g. due to fluctuations in water levels, and rejoin later allowing time for genetic isolation of beetle lineages. Recent comparative phylogeographic analyses of a sympatric sister triplet at Sturt Meadows (aquifer #41; Figure 3) and tree distantly related species at Laverton Downs (aquifer #19; Figure 3), provide some evidence for past population fragmentation events [42]. However, this does not imply that fragmentation is the basis of reproductive isolation between all sympatric sister species. One would expect such processes to occur more often in large or linear aquifers, but there was no difference between the surface area of aquifers containing sister species and aquifers without sister species (t = 0.33, P = 0.74). Sympatric sister species were found in very large, linear aquifers (e.g. Three Rivers aquifer #2: 240 km^2^), as well as in tiny aquifers (e.g. Sons of Gwalia aquifer #35: 2.51 km^2^).

In addition to parapatric/microallopatric speciation, our data do not exclude the possibility of sympatric speciation. Claessen et al. [2] propose sympatric speciation models where cannibalism and competition for food can result in size-structured populations, which can lead to ontogenetic niche shifts and ultimately to evolutionary branching [3]. Interestingly, in the diving beetles, all of the proposed ingredients for such ontogenetic niche shifts were present. The onset of aridity triggered the beetles to take refuge underground. During the transition from the surface to subterranean environment available food sources would have dramatically decreased, leading to fierce competition for food. Furthermore, the diving beetles are at the top of the subterranean food web, as especially larvae of the diving beetles are ferocious predators; and cannibalism among diving beetle larvae is common. In support of Claessen's [3] model, it is noteworthy that the sympatric subterranean species fall into different size categories [24], that are, in most of the localities, significantly non-overlapping (Vergnon et al., in preparation).

To date, studies of sympatric speciation of natural species have been hampered by small numbers of speciation events per taxon, which did not permit ruling out past involvement of geographic barriers to gene flow and repeated colonisation. The only occasions where inferences about geographical distributions of the ancestral species can be made more reliably are where organisms colonized islands [12], [43], including crater lakes [8], [44], or caves [45]. Our data substantially contribute to the study of sympatric evolution, as it demonstrates sympatric sister species in 11 rather than two or three isolated communities, which is unique in that it allowed statistical analysis of the possible speciation modes.

In conclusion, using simple colonization models, we have shown that colonization of aquifers by ancestral diving beetles was largely a random process, and that the high occurrence of sympatric sister species within aquifers is best explained by a process of diversification within the aquifer. Our data thus suggests that within aquifer speciation is not rare in these systems. Due to the large number and variety of speciation events, this group offers considerable potential as a model system for further investigating the factors that promote divergence and speciation.

## Methods

### Taxon sampling and molecular analyses

This research is based on phylogenetic data of 114 diving beetle species belonging to the dytiscid tribes Bidessini and Hydroporini, including 84 subterranean diving beetle species from 45 aquifers in the Yilgarn region of Western Australia and almost all known surface species. We added DNA sequence data of 35 species (mainly from the Bidessini clade) to a mitochondrial DNA data set of 1655 base pairs, which was previously used to study the systematics and evolution of both tribes of diving beetles [21], [39], [46]. DNA methods used are described in Leys & Watts [46]. Uncorrelated lognormal molecular clock analyses with BEAST [47] using a mean rate of 0.0115 substitutions per site per million year [48] and a Yule process of speciation, were performed applying unlinked data partitions for each of the codons for the protein coding genes and separate partitions for stems and loops for RNA genes using a general time reversible model of sequence evolution with invariable sites and gamma distributed rates across sites (GTR+i+g). Tracer v1.4 [49] was used to make sure that the effective sample size (ESS) of the parameters during the BEAST runs were larger than 100. The GenBank accession numbers and estimated parameter values for the examined taxa are given in the supporting information S1 and S2.

We are aware of the potential problems with using a mtDNA tree as a representation of the species tree. However, phylogenetic analyses using the nuclear gene cinnabar [50] and unpublished data, concur with the mtDNA phylogeny presented here, with respect to sympatric sister species relationships. We therefore suggest that our mtDNA phylogeny provides an accurate assessment of the proportion of sympatric sister species and is suitable for testing the modes of colonisation in the subterranean habitats.

### Models of repeated and single colonization of aquifers

The models are based on the assumption that temporary dried up pools in the drainage valleys may fill again after rain, and are recolonized randomly out of a suite of co-occurring diving beetle species. Colonization of the subterranean aquatic habitats would then have taken place at sites where these temporary pools dried out and were connected to calcrete aquifers. The first model was used to test the hypothesis that sympatric sister species may have evolved because colonization of the aquifers had taken place in at least two distinct periods (Figure 2a). The two periods must have been sufficiently far apart to allow for evolution of the first colonizers.

We assume that there are a few niches available in the aquifers (e.g. three in Figure 2a) and that each niche can be occupied by only one species because of competitive exclusion. In the first colonization period (C_1_) each of these niches will be successfully colonized by a random species of a pool of *n* ancestral species with a probability *p_1_*. In a second colonization period (C_2_) the remaining available niches (in Figure 2a represented by the white oval) will be colonized by species randomly drawn from the same species pool with a probability of *p_2_*. In aquifers with two niches, sympatric sister species can only occur through repeated colonization when during the first event only one niche is filled (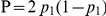) and during the second event the remaining niche is colonized by the same species (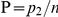). Hence, in aquifers with two niches, the probability of sister species through repeated colonization (P_2n,pair,2c_) is:
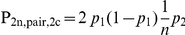
Similarly, in aquifers with three niches, the probability of finding sister species after two colonization events is:
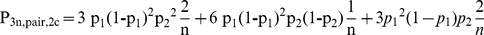
For simplicity we only derive the probability of finding pairs for three colonization events if we assume *p* = *p_1_* = *p_2_* and *p_3_* = 1. The probability of finding pairs in two niches after three colonization events is then:
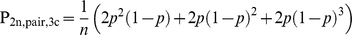
The probability of finding pairs in aquifers with three niches after three events can be derived as:
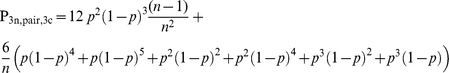
Triplets can only occur through independent colonizations if we assume that there are at least three successful colonization events. The probability of finding triplets through independent colonizations is then:
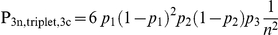
To calculate the maximum overall expected fraction of aquifers containing sister species pairs due to repeated colonization, we assume that the current number of species reflects the number of niches in an aquifer, and therefore, that all available niches become occupied during the last colonization. After two colonization periods, the maximum expected fraction of aquifers that contain sister pairs is then:
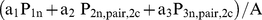
(1)And for three colonization periods:
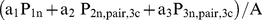
(2)Where P_1_ = 0, A is the total number of aquifers, and a_1_–a_3_ are the number of aquifers with 1–3 species. The expected fraction of aquifers containing triplets is
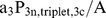
(3)In a second model we test the hypothesis that sympatric sister species are the result of a single colonization per aquifer. Here, after a colonization event with probability *p*, it is assumed that remaining empty niches are filled following diversification of a species that previously colonized a different niche in the aquifer with a probability of *q* (species 2 in Figure 2b). Thus, for single colonization events the probabilities of finding sister species pairs for aquifers with 2–3 niches are:
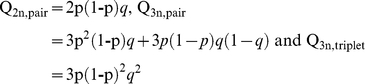
Note that the probability of sympatric sister species occurring for both single colonization and within aquifer divergence does not depend on the number of ancestral species, and that it is possible for aquifers that have three niches to obtain a triplet of sympatric sister species when only one niche is colonized initially.

The overall expected fraction of sister species pairs arising by speciation within aquifers is:
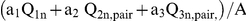
where A is the total number of aquifers and a_1–3_ are the number of aquifers with 1–3 niches. We analyzed only the extreme case in which all remaining niches are filled following diversification (*q* = 1).

We based the number of niches a_1–3_ for each aquifer on the total number of recorded species per aquifer [24]. This approach assumes that after the last colonization there are no empty niches in the observed aquifers, while in the model this may occur. To be able to compare the modeled and observed values without this bias, we assumed that in the last colonization event all niches were filled (p_2_ = 1 respectively p_3_ = 1), an assumption that leads to overestimation of the number of sister species (supporting information S3). We tested the behaviour of the model for different values of initial colonization probability and initial sizes of the ancestral species pool (supporting information S3). Based on this analysis, we chose our colonization probablilities to maximize the expected number of aquifers containing species pairs. We generated confidence limits by drawing the species of the 45 aquifers at random using the described models. The 5 and 95 percentiles of 10000 repetitions were used as the confidence limits. The randomisation program is available from the corresponding author.

### Assumption of the models

Our repeated colonization model relies on the following assumptions:

(A) The ancestral species have an equal chance to make a successful transition into an aquifer. To meet this assumption the ancestral species must have had largely overlapping geographical distributions, which is supported by the available data, see discussion. (B) We take the number of species presently found in each aquifer to reflect the number of species that can colonise these aquifers. For this model we assume that each species occupies a single niche. (C) We consider the probability that a niche becomes occupied as the positive end-result of a range of processes that eventually leads to the occupation of a niche. We presume that when a niche is not occupied in a first colonization it will be in a second colonization period. These processes may also include initial colonization of a waterhole by surface species, surviving local competition, moving to the subsurface (interstitial) habitat during drying of the surface water and finally colonizing a subterranean niche.

## Supporting Information

Supporting Information S1 List of examined taxa with details on sample localities, and GenBank accession numbers.
(XLS)

Supporting Information S2 Parameter estimates of the BEAST analyses calculated using Tracer.
(DOC)

Supporting Information S3 The relationship between the size of the ancestral species pool, niche colonization probabilities and the fraction of aquifers with sister species calculated with two and three colonization events.
(DOC)
