# Supplementary figures and images for: Evolution of Blind Beetles in Isolated Aquifers: A Test of Alternative Modes of Speciation
Source: PLoS One. 2012 Mar 30;7(3):e34260. doi: 10.1371/journal.pone.0034260 (PMC3316697; doi:10.1371/journal.pone.0034260)

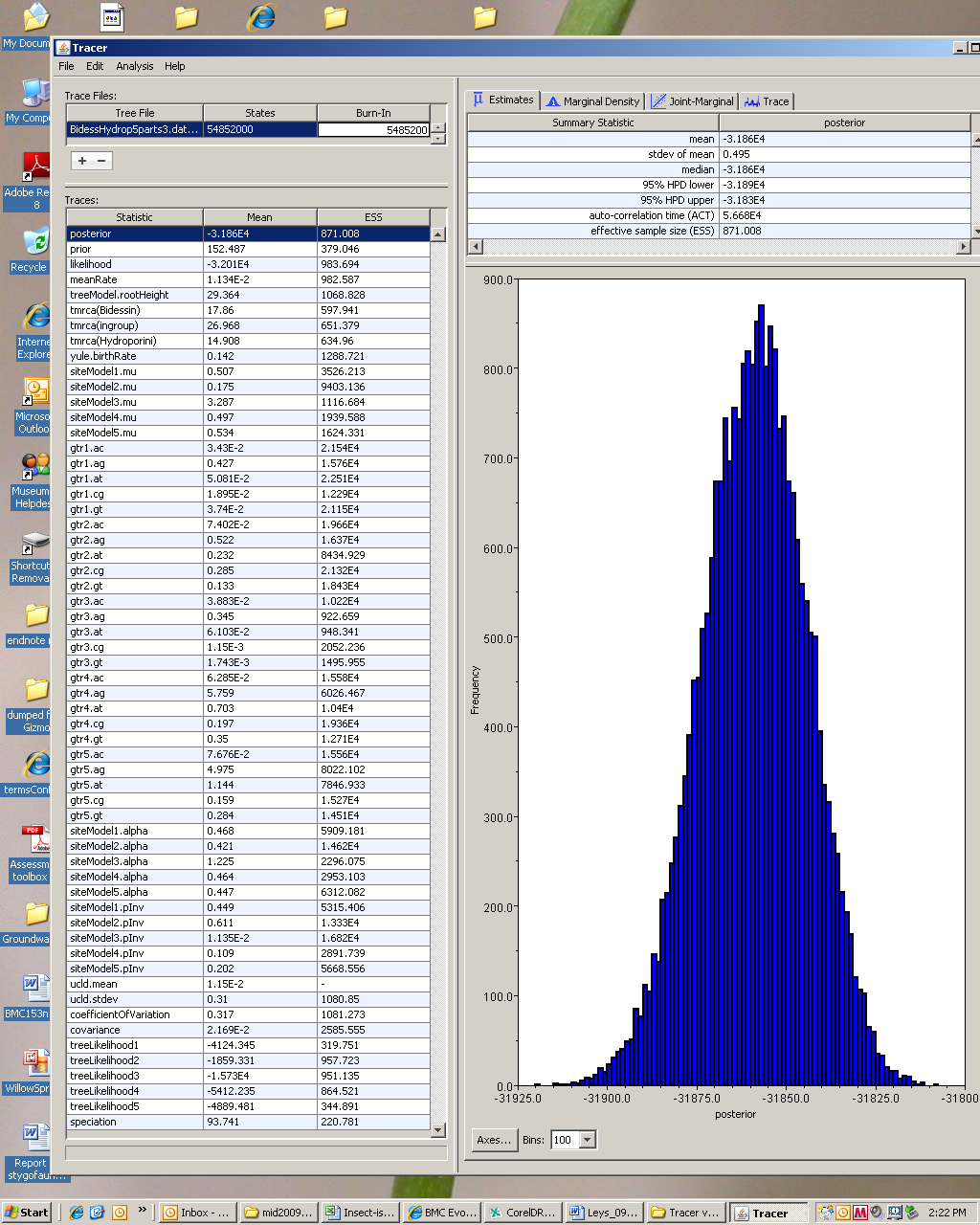

Supplement: Supporting Information S2 — Parameter estimates of the BEAST analyses calculated using Tracer. (DOC) [file pone.0034260.s002.doc]
