# Supplementary material for: Evolution of Blind Beetles in Isolated Aquifers: A Test of Alternative Modes of Speciation
Source: PLoS One. 2012 Mar 30;7(3):e34260. doi: 10.1371/journal.pone.0034260 (PMC3316697; doi:10.1371/journal.pone.0034260)

**Supporting Information S3**

The relationship between the size of the ancestral species pool, niche colonization probabilities (*p*) and the fraction of aquifers with sister species calculated for (A) aquifers with two niches, (B) three niches - sister pairs, and (C) three niches - sister triplets. The figures on the left show the relationships where *p* is the same for each subsequent colonization. The right hand figures show the relationships where *p*=1 in the last colonization in order to fill up all remaining empty niches. Note that the latter approach leads to a higher expected fraction of aquifers with sister pairs. Thus, in our analysis of the repeated colonisation model (Fig 4a and b in the main text), we maximized the probabilities to obtain sister species by chosing *p*1=0.5 and *p*2 =1 for two colonizations and *p*1=*p*2=0.4 and *p*3=1 for three colonizations. Note also that both approaches lead to extremely small probabilities of sister triplets as a result of multiple colonizations (C).


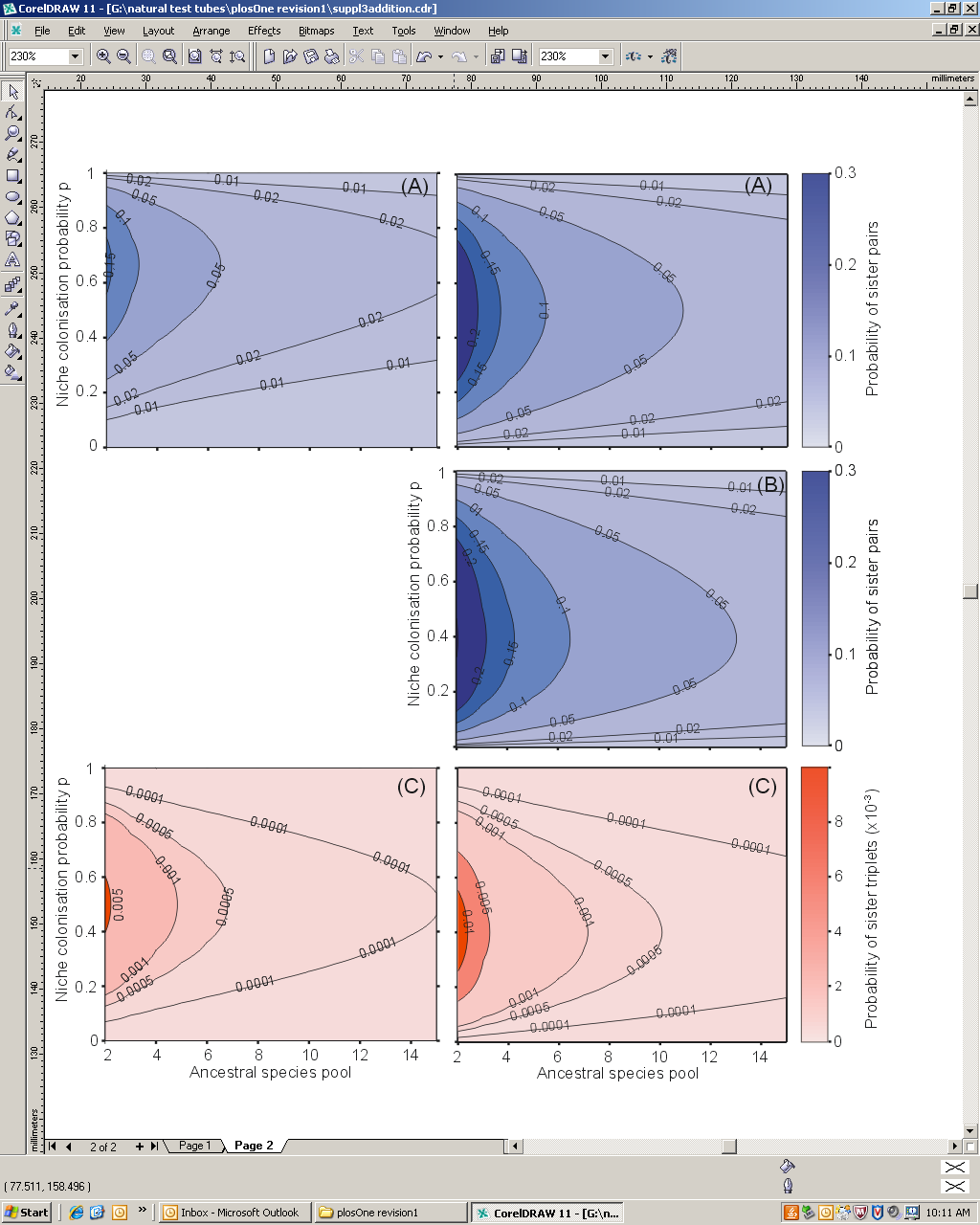

Supplement: Supporting Information S3 — The relationship between the size of the ancestral species pool, niche colonization probabilities and the fraction of aquifers with sister species calculated with two and three colonization events. (DOC) [file pone.0034260.s003.doc]
